# Atopy as an independent predictor for long-term patient and graft survival after kidney transplantation

**DOI:** 10.3389/fimmu.2022.997364

**Published:** 2022-10-03

**Authors:** Raphaël Porret, Raphaël P. H. Meier, Josip Mikulic, Manuel Pascual, Vincent Aubert, Thomas Harr, Déla Golshayan, Yannick D. Muller

**Affiliations:** ^1^ Division of Immunology and Allergy, Centre Hospitalier Universitaire Vaudois and University of Lausanne, Lausanne, Switzerland; ^2^ Department of Surgery, University of Maryland School of Medicine, Baltimore, MD, United States; ^3^ Transplantation Center, Centre Hospitalier Universitaire Vaudois and University of Lausanne, Lausanne, Switzerland; ^4^ Division of Immunology and Allergy, University Hospitals of Geneva, Geneva, Switzerland

**Keywords:** atopy, transplantation, kidney, survival, rejection, graft survival, patient survival

## Abstract

**Background:**

Atopy is a genetic condition predisposing individuals to develop immunoglobulin E (IgE) against common allergens through T-helper 2 (Th2) polarization mechanisms. The impact of atopy on graft survival in solid organ transplantation is unknown.

**Methodology:**

We analyzed 268 renal allograft recipients from the Swiss Transplant Cohort Study, a prospective multicenter cohort studying patients after solid organ transplantation, with a 9-year median follow-up (IQR 3.0). We used the Phadiatop assay to measure IgE antibodies against a mixture of common inhaled allergens (grass, tree, herbs, spores, animals, and mites) to identify pre-transplantation atopic patients (>0.35 KU/L).

**Results:**

Of 268 kidney transplant recipients, 66 individuals were atopic (24.6%). Atopic patients were significantly younger than non-atopic patients (49.6 vs 58.0 years old, P = 0.002). No significant difference was found for gender, cold/warm ischemia time, preformed donor-specific antibodies (DSA), HLA mismatches, induction and maintenance immunosuppressive therapy, CMV serostatus, or cause of kidney failure. Patient and graft survival at ten years of follow-up were significantly better in the atopic group, 95.2% versus 69.2% patient survival (P < 0.001), and 87.9% versus 60.8% graft survival (P < 0.001), respectively. A multivariate Cox analysis revealed that atopy predicted recipient and graft survival independently of age and living donor donation. Finally, we found similar rates of biopsy-proven acute cellular and antibody-mediated rejections between atopic and non-atopic recipients.

**Conclusion:**

Atopy was associated with better long-term patient and graft survival, independently of age and living donor donation after kidney transplantation. Yet, atopy should not be used as a predictor for acute rejection.

## Introduction

The T helper 1/T helper 2 (Th1/Th2) balance is a key element involved in regulation of various immunological responses, such as host defense against microorganisms, autoimmune diseases or allergy. Appreciation of its complexity has substantially increased since the discovery of various other T helper cell subsets, including Th17, T follicular helper (Tfh), or regulatory T cells, as well as with the recognition of the interplay with the innate immune system (1). Th1 cells drive a predominant cellular cytotoxic immunity mediated by IL-12/IFN-γ, whereas Th2 cells facilitate IL-4/IL-5-mediated humoral responses, notably through immunoglobulin E (IgE) production and eosinophilia (2). The genetic background predisposes individuals to develop a predominant T helper polarization, possibly resulting in allergy development, autoimmunity, or in parasite resistance (3) (4).

The genetic predisposition for the development of specific IgE against common allergens, such as pollens, dusts, or animals defining atopy may be associated with rhino-conjunctivitis, asthma, atopic dermatitis and food allergies (5). Atopic patients produce aberrant specific IgE antibodies, possibly associated with eosinophilia, which is the hallmark of a predominant Th2 response (6). Based on the Swiss Study on Air Pollution and Lung Diseases in Adult (SAPALDIA), the prevalence of atopy was estimated to be around 30% in younger adults (<60 years old) and 20% in older adults (>60 years old) (7). Thus, atopic individuals may represent a substantial number of patients in organ transplantation, both as donors or recipients. Yet, there are no solid data on the relationship between atopy and allograft survival.

The primary objective of this study was to compare kidney allograft and patient survival, between atopic and non-atopic recipients in a Swiss prospective multicenter cohort. We defined atopy by the presence of a positive Phadiatop prior to transplantation, as the latter has a high sensitivity (92%) and specificity (98%) (8). We then compared the occurrence of acute cellular and antibody-mediated rejection episodes in both groups, i.e. atopic vs non-atopic individuals.

## Methods

### Study design

All patients were enrolled in the Swiss Transplant Cohort Study (STCS), a prospective multicenter cohort, including all solid organ recipients at the Swiss national level as of May 2008 onwards (9, 10). 268 sera with sufficient available amount for analysis in Phadiatop could be retrieved from a total of 710 consecutive samples (from May 2008 to 2011) allocated to a separate study (11). All recipients were Caucasians and received a kidney transplantation between May 2008 and October 2015, with regular follow-up until December 2020, or until graft failure or patient’s death. 260 patients underwent a kidney-alone transplantation, and four patients received two en-bloc kidneys. Two patients were retransplanted with a second kidney in 2015; their data were collected as two independent follow-ups. This study was approved by the local ethical committee (CCER, ID-2017-01032), as well as by the STCS (FUP 098). All patients signed a written informed consent.

### Data collection

Data were collected at day 0 before transplantation, 6 and 12 months post transplantation, and then every year, using standardized follow-up forms. Baseline donor and recipient characteristics included ethnicity, age and gender, HLA mismatches, anti-HLA antibodies and pre-formed donor-specific antibodies (DSA), CMV serostatus, as well as recipient diagnosis of end-stage renal disease and pre-existing co-morbidities. Transplantation-related characteristics included type of donor, cold and warm ischemia time, induction and initial maintenance immunosuppressive therapy, and surgery-related complications. Recipient follow-up data included graft loss and rejection status (biopsy-proven acute rejection episodes and corresponding Banff classification) (12), BK viremia, CMV infections, anti-HLA antibodies and DSA tests results, serum creatinine, and proteinuria.

### Phadiatop and IgE analysis

Analyzes were performed retrospectively on frozen serum samples, obtained on the day of transplantation. Specific IgE were measured using ImmunoCAP technology (Phadia 250, Thermo Fischer Scientific, Waltham, Massachusetts). The Phadiatop is a test quantifying IgE against a mixture of common respiratory allergens including grass, birch, olive, mugwort, parietaria, dog, cat, horse, house dust mite and flour mite, and *Cladosporium* (7). Total IgE concentrations in plasma samples were analyzed by ImmunoCAP (Pharmacia Diagnostics, Uppsala, Sweden) according to the manufacturer’s instructions. The lower detection limit was 0.35 kU/L for the Phadiatop assay, and 2 kU/L for the conventional total IgE assay. Total IgE values were used as a continuous variable for comparing atopic and non-atopic patients while the Phadiatop was used as a categorical variable. Patients with a positive Phadiatop (≥ 0.35 kU/L) were considered atopic.

### Statistical analysis

All statistical analysis were performed using R environment (version 4.0.4). A p-value smaller than 0.05 was considered to be statistically significant. Differences between groups were assessed by a Mann-Whitney test for continuous variables, and a Chi-squared test for proportions. Pearson’s Chi-squared test was used for categorical variables. Survival analyses were performed with the Kaplan–Meier method and the log rank test. Deaths with a functioning graft were not recorded in this study and were considered as graft loss. Multivariate Cox proportional-hazards regression analysis was used to adjust for the recipient’s age, non-living donation, and long cold ischemia time. Univariate Cox regression model was computed for each independent variable, using the *coxph()* function of the survival package. Multivariate analysis was performed by using stratified multivariable Cox model. Wald test was used to assess significance in Cox proportional hazards regression analysis. Bar plots were generated using the *ggpubr* package.

## Results

### Study population

Two hundred sixty-eight Caucasian kidney transplant recipients from the STCS were included in this study. Median follow-up was 8.8 years (interquartile range (IQR) 4.1) for the non-atopic and 9.0 years (IQR 2.0) for the atopic group. Their baseline characteristics are described in **Table 1**. 262 patients (97.8%) were transplanted for the first time, whereas six of the total patient cohort already had a previous kidney transplant, or had undergone double transplantation. Considering that immunosuppression has a little/no impact on antibodies productions in the context of allergy transfer but also in individuals suffering from seasonal rhinoconjunctivitis (13), we didn’t exclude those individuals from the analysis. The majority of the organs were obtained from brain dead donors (60.8%).

**Table 1:** Characteristics of non-atopic and atopic kidney transplant recipients at the time of transplantation.

Variable	Non-atopic		Atopic		
		*N = 202*		*N = 66*		* *
	N (% or IQR)		N (% or IQR)		P
Donor age (median years; IQR)	54.5	(19.0)	51.5	(15.8)	0.29
Donor gender M	108	(53.5%)	28	(42.4%)	0.12
Recipient age (median years; IQR)	57.4	(18.7)	49.0	(21.3)	**0.002**
Recipient gender M	137	(67.8%)	50	(75.8%)	0.22
Type of donor					
Living related donor	42	(20.8%)	20	(30.3%)	0.11
Living unrelated donor	28	(13.9%)	15	(22.7%)	0.09
Cold ischemia time (median hours; IQR)^a^	7.1	(8.3)	6.3	(9.5)	0.09
Warm ischemia time (median minutes; IQR)^b^	5.2	(3.0)	5.3	(4.0)	0.11
Multiple kidney transplants	5	(2.5%)	1	(1.5%)	0.65
HLA mismatches (>3)^c^	114	(56.7%)	36	(54.5%)	0.76
HLA-A mismatches (0/1/2)^d^	44/89/68	(21.9%/44.3%/33.8%)	13/30/23	(19.7%/45.5%/34.8%)	
HLA-B mismatches (0/1/2)	21/89/92	(10.4%/44.1%/45.6%)	8/27/31	(12.1%/40.9%/47.0%)	
HLA-DR mismatches (0/1/2)	24/113/65	(11.9%/55.9%/32.2%)	10/32/24	(15.2%/48.5%/36.4%)	
DSA					
Preformed DSA^e^	39	(50.6%)	6	(31.6%)	0.14
Donor/recipient CMV serostatus^f^					
CMV D-R-	46	(22.9%)	23	(35.8%)	0.05
CMV D+R-	41	(20.4%)	11	(16.7%)	0.51
CMV D-R+	41	(20.4%)	12	(18.2%)	0.70
CMV D+R+	73	(36.3%)	20	(30.3%)	0.37
Induction therapy^g^					
Basiliximab only	141	(70.8%)	51	(80.9%)	0.11
Anti-thymocyte globulin only	11	(5.5%)	2	(3.2%)	0.45
Basiliximab + ATG	2	(1.0%)	2	(3.2%)	0.22
Basiliximab + ATG + IVIG	1	(0.5%)	0	(0.0%)	0.57
Basiliximab + ABO-Immunoadsorption	4	(2.0%)	1	(1.6%)	0.83
ATG + IVIG	39	(19.6%)	7	(11.1%)	0.12
ATG + Plasmapheresis	1	(0.5%)	0	(0.0%)	0.57
Initial maintenance immunosuppression					
Corticosteroids	200	(99.0%)	65	(98.5%)	0.73
Tacrolimus	162	(80.2%)	52	(78.8%)	0.80
Cyclosporine	40	(19.8%)	14	(21.2%)	0.80
MPA agents	197	(97.5%)	64	(97.0%)	0.81
AZA	2	(1.0%)	1	(1.5%)	0.73
mTOR inhibitors	20	(9.9%)	9	(13.6%)	0.40
Other maintenance therapy	3	(1.5%)	0	(0.0%)	0.32
Atopic status					
IgE total (median; IQR)	15.3	(28.7)	89.4	(142.3)	**< 0.001**

ATG, anti-thymocyte globulin; AZA, azathioprine; CMV, cytomegalovirus; D, donor; DSA, donor-specific antibody; HLA, human leukocyte antigen; Ig, immunoglobulin; IQR, interquartile range; IVIG, intravenous immunoglobulin; M, male; MPA, mycophenolic acid; mTOR, mammalian target of rapamycin; P, p-value; R, recipient.

p-values were computed using a Welch two sample t-test for continuous variables, and a two-sample test for equality of proportions without continuity correction for percentages.

a Data were missing for 4 patients in the non-atopic group, and for 2 patients in the atopic group.

b Data were missing for 12 patients in the non-atopic group, and for 1 patient in the atopic group.

c Data were missing for 1 patient in the non-atopic group.

d Data were missing for 1 patient in the non-atopic group.

e Data were missing for 125 patient in the non-atopic group, and for 47 patients in the atopic group.

f Data were missing for 1 patient in the non-atopic group.

g Data were missing for 3 patients in the non-atopic group and for 3 patients in the atopic group. Bold values are statistically significant values (P <= 0.05).

66 transplant recipients (24.6%) had a positive Phadiatop. Atopic recipients were significantly younger than non-atopic recipients (49.0 vs 57.4 years old, P = 0.002) and had significantly higher levels of total IgE (89.4 vs 15.3, P < 0.001, **Table 1**). No significant differences were found between non-atopic and atopic groups for donor age, donor gender, recipient gender, type of donor (living related versus unrelated), cold and warm ischemia time, HLA mismatches, presence of DSA at the time of transplantation, donor/recipient CMV serostatus, induction and maintenance immunosuppressive therapy. No statistically significant differences were found between the two groups regarding end-stage renal disease causes (P = 0.56, **Supplementary Table 1**) and transplantation surgery-related complications (P = 0.33, **Supplementary Table 2**). The incidence of BK viremia and nephritis at one year post transplantation was comparable between the non-atopic and the atopic group (12.3% vs 16.0% respectively, P = 0.89). There was no difference either in the incidence of CMV infections between both groups during the entire follow-up period (36.6% vs 28.8%, P = 0.31).

### Patient and graft survival

**Figure 1:**
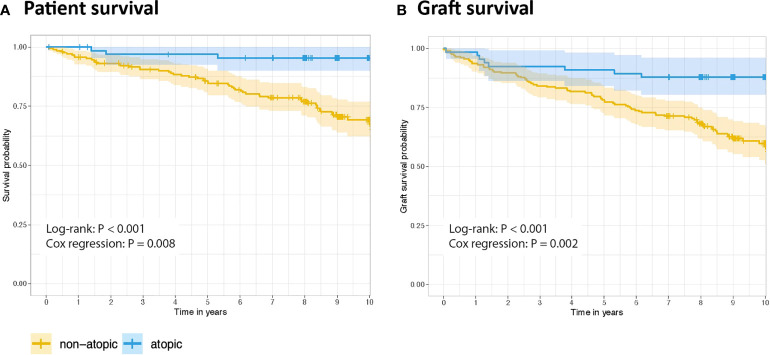
Kaplan-Meier estimates of ten-year follow-up. **(A)** Patient survival, and **(B)** kidney graft survival. The calculated p-values were obtained by the log-rank test (univariate analysis). Age-adjusted p-values were calculated with multivariate Cox proportional hazards regression analysis. 95% confidence intervals are represented according to the group color.

**Figure 2:**
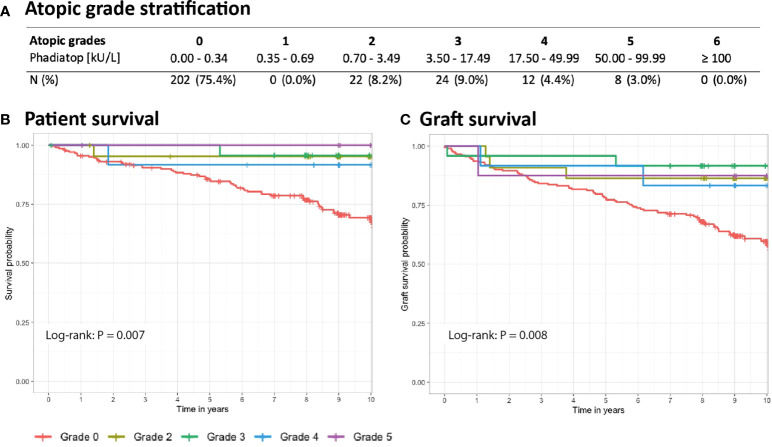
Kaplan-Meier estimates of ten-year follow-up stratified by atopic grade. **(A)** Number of patients in each subgroup based on Phadiatop grading. **(B)** Patient survival and **(C)** graft survival across all groups. The calculated p-values were obtained by the log-rank test (univariate analysis).

One-year overall patient survival was 100% and 95.5% in the atopic and non-atopic group (P = 0.085), whereas graft survival was 98.5% and 93.6.0%, respectively (P = 0.12). Long-term patient survival was significantly better in the atopic group (95.2% versus 69.2%, P < 0.001, **Figure 1A**). When adjusted to recipient age, patient survival of the atopic group remained significantly better at 10 years as compared to the non-atopic group (93.5% versus 76.6%, P = 0.008). Similarly, graft survival at ten years was 87.9% and 59.7% in the atopic and non-atopic group, respectively (P < 0.001, **Figure 1B**). This difference remained statistically significant when adjusting for the recipient age (85.4% versus 62.1%, P = 0.002). We further analyzed patient and graft survival stratifying the Phadiatop results from grade 0 (negative) to six (strongly positive, **Figure 2A**). In the atopic group, the graft and patient survival was independent of the phadiatop grading (**Figures 2B, C**).

As patient’s age could affect both total IgE and specific IgE serum concentration (14), we further stratified our study population based on recipient’s age with a cut-off at 50-year-old (**Supplementary Table 3**). In the < 50-year-old group, 35.1% (33/94) were atopic against 19% (33/174) in >50-year-old group (P = 0.005). However, no differences in total IgE levels between both groups were observed, possibly by lack of statistical power (P = 0.12). We next compared atopic and non-atopic graft and patient survival in both age groups (<50- and >50-year-old). While the ten-year patient survival was not statistically significant in the group <50 (P = 0.287), the graft survival was close to significancy (P = 0.055) in atopic patients. Importantly, in the group >50, the ten-year patient (90.3% and 60.8%, p = 0.004) and graft (84.8% and 55.1%, P = 0.007) survival was significantly lower in non-atopic patients reinforcing the results of the multivariate analysis showing atopy as an independent predictive factor of graft and patient survival (**Supplementary Figure 2**).

### Confounding factors

In contrast to atopy being positively associated with patient survival and graft survival, our univariate analysis revealed that patient survival was negatively influenced by recipient age, non-living donation and long cold ischemia time (**Table 2**). We therefore next performed a multivariate analysis, using a stratified Cox model. We found that among the factors that negatively influenced patient survival, atopy was an independent protective factor from death (hazard ratio [HR] 0.31, 95% confidence interval [CI] 0.11-0.88, P = 0.027, **Table 2**). Living donor (HR 0.27, 95% CI 0.11-0.66, P = 0.004) and recipient age (HR 1.05, 95% CI 1.03-1.08, P < 0.001), but not cold ischemia time (HR 0.97, 95% CI 0.90-1.04, P= 0.34), were independent predictors of long-term outcome. Similarly, the univariate Cox analysis for graft survival revealed recipient age, living donation, and cold ischemia time as influencing factors (**Table 3**). Multivariate Cox regression analysis confirmed that atopy was also an independent protective factor from graft loss (hazard ratio [HR] 0.36, 95% confidence interval [CI] 0.17-0.75, P = 0.006, **Table 3**). Living donor was another independent protective factor (HR 0.24, 95% CI 0.11-0.52, P < 0.001), but neither recipient age (HR 1.01, 95% CI 1.00-1.03, P = 0.15), nor cold ischemia time (HR 0.97, 95% CI 0.91-1.04, P = 0.37) were independently linked to graft loss.

**Table 2:** Risk factors for patient mortality during follow-up.

Variable	Univariate Cox model		Multivariate Cox model	
	HR (95% CI)	P	HR (95% CI)	P
Donor age	1 (0.99-1)	0.17		
Donor female gender	0.83 (0.5-1.4)	0.45		
Recipient age	1.1 (1-1.1)	**<0.001**	1.05 (1.03-1.08)	**<0.001**
Recipient female gender	0.97 (0.57-1.7)	0.91		
Living donor	0.27 (0.14-0.51)	**<0.001**	0.27 (0.11-0.66)	**0.004**
Cold ischemia time	1.1 (1-1.1)	**0.0053**	0.97 (0.90-1.04)	0.34
Warm ischemia time	1 (0.99-1)	0.35		
HLA mismatches (> 3)	1.4 (0.86-2.4)	0.17		
Preformed DSA	1.1 (0.46-2.5)	0.88		
Rejection episodes (regrouping ACR and AMR)	1 (0.63-1.7)	0.89		
Donor/recipient CMV status				
CMV D-R-	reference			
CMV D+R-	0.99 (0.48-2)	0.97		
CMV D-R+	0.49 (0.21-1.1)	0.10		
CMV D+R+	1 (0.56-1.9)	0.9		
Induction therapy				
Basiliximab only	1.2 (0.67-2.2)	0.51		
ATG only	0.72 (0.18-3)	0.65		
Maintenance therapy				
Steroids	0.54 (0.075-3.9)	0.55		
Tacrolimus	0.81 (0.43-1.5)	0.51		
Cyclosporine	1.1 (0.56-2.1)	0.81		
MPA agents	0.68 (0.17-2.8)	0.60		
AZA	1.3 (0.18-9.3)	0.80		
mTOR inhibitors	0.89 (0.42-1.9)	0.75		
ATG	3e-07 (0–)	1		
Atopic status				
IgE all	1 (1-1)	0.27		
Positive phadiatop	0.18 (0.07-0.5)	**<0.001**	0.31 (0.11-0.88)	**0.027**

ACR, acute cellular rejection; AMR, acute antibody-mediated rejection; ATG, anti-thymocyte globulin; AZA, azathioprine; CI, confidence interval; CMV, cytomegalovirus; D, donor; DSA, donor-specific antibody; HLA, human leukocyte antigen; HR, hazard ratio; Ig, immunoglobulin; IVIG, intravenous immunoglobulin; M, male; MPA, mycophenolic acid; mTOR, mammalian target of rapamycin; P, p-value; R, recipient. Bold values are statistically significant values (P <= 0.05).

**Table 3:** Risk factors for graft loss during follow-up.

Variable	Univariate Cox model		Multivariate Cox model	
	HR (95% CI)	P	HR (95% CI)	P
Donor age	1 (1-1)	0.08		
Donor female gender	0.88 (0.58-1.3)	0.55		
Recipient age	1 (1-1)	**0.0073**	1.01 (1.00-1.03)	0.15
Recipient female gender	0.78 (0.48-1.2)	0.29		
Living donor	0.27 (0.16-0.47)	**<0.001**	0.24 (0.11-0.52)	**<0.001**
Cold ischemia time	1.1 (1-1.1)	**<0.001**	0.97 (0.91-1.04)	0.37
Warm ischemia time	1 (0.99-1)	0.59		
HLA mismatches (> 3)	1.4 (0.89-2.1)	0.16		
Preformed DSA	1.2 (0.63-2.2)	0.62		
Rejection episodes (regrouping ACR and AMR)	1.2 (0.8-1.8)	0.35		
Donor/recipient CMV status				
CMV D-R-	reference			
CMV D+R-	0.9 (0.48-1.7)	0.75		
CMV D-R+	0.66 (0.34-1.3)	0.22		
CMV D+R+	1.2 (0.7-2)	0.53		
Induction therapy				
Basiliximab only	0.82 (0.52-1.3)	0.39		
ATG only	1.2 (0.5-3)	0.65		
Maintenance therapy				
Steroids	0.8 (0.11-5.7)	0.82		
Tacrolimus	0.67 (0.41-1.1)	0.12		
Cyclosporine	1.4 (0.81-2.3)	0.24		
MPA agents	1 (0.26-4.2)	0.96		
AZA	0.93 (0.13-6.7)	0.94		
mTOR inhibitors	0.61 (0.29-1.3)	0.18		
ATG	2.5 (0.62-10)	0.2		
Atopic status				
IgE all	1 (1-1)	0.18		
Positive phadiatop	0.3 (0.15-0.59)	**<0.001**	0.36 (0.17-0.75)	**0.006**

ACR, acute cellular rejection; AMR, acute antibody-mediated rejection; ATG, anti-thymocyte globulin; AZA, azathioprine; CI, confidence interval; CMV, cytomegalovirus; D, donor; DSA, donor-specific antibody; HLA, human leukocyte antigen; HR, hazard ratio; Ig, immunoglobulin; IVIG, intravenous immunoglobulin; M, male; MPA, mycophenolic acid; mTOR, mammalian target of rapamycin; P, p-value; R, recipient. Bold values are statistically significant values (P <= 0.05).

Preformed and *de novo* donor specific antibodies could represent a confounding factor in this analysis. At the time of transplantation, the presence of preformed DSA were reported only in 36% of the cases. Cumulative MFI was not significantly different (mean +/- SD = 3023 +/- 3228 for the atopic group and 6068 +/- 9295 for the non-atopic group). When excluding individuals with preformed antibodies from the analysis, difference between atopic and non-atopic patient and graft survivals remains statistically significant (P < 0.001). Additionally, we did not find any significant correlation between total IgE and cumulative DSA (**Supplementary Figure 1**). Finally, to estimate the probability of developing *de novo* DSA, we identified 13 atopic and 46 non-atopic patients who had no preformed DSA at baseline. Twenty of them had detectable DSA during follow-up, out of which four were atopic (20%) and 16 non-atopic (80%). DSA MFI were however not reported limiting the interpretation of the results.

### Biopsy-proven rejection episodes in atopic and non-atopic groups

**Figure 3:**
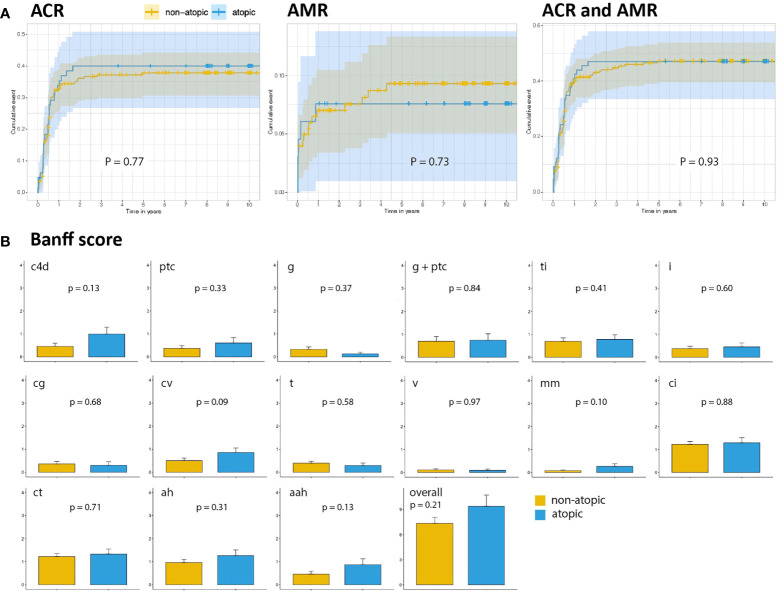
Biopsy-proven rejections in atopic and non-atopic groups. **(A)** Ten-year cumulative events of ACR and AMR according to the atopic status. **(B)** Banff score comparison of kidney biopsies in non-atopic and in atopic patients. Bars represent the average score of all biopsies performed in the non-atopic and atopic patients during follow-up. Intervals represent the standard error of the mean. C4d, C4d deposition; ptc, peritubular capillaritis; g, glomerulitis; ti, total inflammation; i, interstitial inflammation; cg, glomerulopathy; cv, fibrous intimal thickening; t, tubulitis; v, intimal arteritis; mm, mesangial matrix increase; ci, interstitial fibrosis; ct, tubular atrophy; ah, arteriolar hyalinosis; aah, alternative arteriolar hyalinosis.

To understand if the differences in graft survival were related to rejection episodes, we next studied the cumulative events of biopsy-proven acute cellular rejection (ACR) or acute antibody-mediated rejection (AMR) in both groups. One-year ACR cumulative hazard for non-atopic and atopic patients were 32.8% versus 33.8% respectively for ACR (P = 0.89), and 7.0% versus 7.6% (P = 0.86) for AMR (**Figure 3A**). When considering overall acute rejection cumulative events (ACR and AMR) at one-year follow-up, we did not find significant differences between both groups either, with 39.8% for non-atopic and 40.9% for atopic patients (P = 0.85, **Figure 3A**). ACR and/or AMR cumulative hazards remained comparable between both groups at ten-year follow-up (**Figure 3A**).

We then analyzed the Banff scores independently of their timing after transplantation or of the rejection pattern. Active AMR-associated histological lesions, including capillary complement 4d (C4d) deposits and the microvascular inflammation score, were assessed by combining glomerulitis and peritubular capillaritis scores. No differences were observed between both groups (P = 0.92, **Figure 3B**). Chronic lesion scores, including chronic glomerulopathy, peritubular capillary lamellation, and chronic arteriolopathy, did not differ between groups either (**Figure 3B**). Similarly, there was no difference in the degree of inflammation, tubulointerstitial infiltrates, or interstitial fibrosis and tubular atrophy.

## Discussion

To the best of our knowledge, this is the first study investigating the role of atopy in a large set of kidney transplant recipients. Here, we investigated the relationship between atopy and patient or kidney graft survival, and found that atopy is an independent protective factor for patient survival 10 years after kidney transplantation. Atopy also correlated with long-term graft survival.

The rationale for performing this study was driven by the experimental observation that graft survival is different whether a Th1 polarized mouse strain is used as a recipient or donor and inversely (15). For example, C57BL/6 mice are imprinted with a genetic background favoring Th1 polarization, INF-γ and IL-2 secretion, thereby promoting alloreactive immune responses and graft rejection. On the other hand, BALB/C mice develop a predominant Th2 response, producing IL-4, IL-5, IL-6, favoring IgE production and eosinophilia (16–19). These differences have been linked to the survival of allogeneic pancreatic islets, which is shorter when BALB/C islets are transplanted into Th1-inflammatory prone C57BL/6 mice, compared to the reverse combination with C57BL/6 islets transplanted into BALB/c mice (15, 20, 21).

Our data show that atopy has no significant impact on short-term graft outcome, as the number and severity of acute rejection episodes one-year post transplantation were similar between both groups. Instead, atopy appears as an independent predictor of long-term patient and graft survival, independently of the Phadiatop grading. It is tempting to postulate that atopy protects patients from chronic allograft dysfunction, although we have no data to support this hypothesis. Thus, the analysis for preformed and *de novo* DSA remained limited. In future studies, it would therefore be important to systematically compare the presence of chronic lesions in the biopsies of atopic and non-atopic patients at later time-points and correlate these results with the presence of DSA (22).

Importantly, we have recently shown that immunosuppressive drugs only marginally affect IgE-sensitization and allergic symptoms in atopic kidney recipients (13). This is further supported by several reports, demonstrating newly acquired peanut-allergy as early as five days post-transplantation and lasting up to seven years (23–25). Also, immunosuppression does not prevent the development of allergies in children receiving solid organ transplant (26–28). These results corroborate older data showing that previously non-asthmatic lung transplant recipients can become asthmatic if they receive the lungs from an asthmatic donor (29). Altogether, these results reinforce the notion that atopy is a genetic predisposition that is poorly affected by immunosuppressive drugs and therefore could represent an independent predictor for patient and graft survival.

Our study has limitations, the main one being the limited number of participants which could be included in the analysis, i.e. a total of 66 atopic patients. Thus, this might have impeded statistically significant observations and could be associated with type 2 errors. Another important limitation is that we could not identify the cause for the prolonged graft survival in atopic patients. Additionally, we could not investigate the cause of death of the recipients, as these data were not available. Finally, a more careful monitoring of DSA over time could also help to dissect the mechanisms responsible for the prolonged graft survival observed in atopic patients. Thus, our results need to be validated in a larger and independent cohort of kidney transplant recipients.

In conclusion, based on a 10-year follow-up, we found that atopy was associated with a better long-term patient and graft survival in kidney transplantation. Further studies are needed to confirm and investigate the mechanisms responsible for this interesting observation, including a more detailed analysis of the immune ecosystem (intra-graft innate response/Th2 cytokine milieu). Unlike mouse models however, atopic patients were not better protected against acute rejection episodes. This suggests that atopy should not be used as an early marker to predict short-term graft outcomes, nor to modulate immunosuppressive protocols.

## Data availability statement

The raw data supporting the conclusions of this article will be made available by the authors, without undue reservation.

## Ethics statement

This study was reviewed and approved by the ethical committee (CCER, ID-2017-01032), as well by the STCS (FUP 098). The patients/participants provided their written informed consent to participate in this study.

## Author contributions

YM designed the study. RP and YM wrote of the manuscript. RP, RM, and YM analyzed the data. JM and DG identified the sera. VA performed the allergic testing. MP, TH, and DG reviewed and edited the manuscript. All authors have read and approved the final version of the manuscript.

## Appendix

The members of the Swiss Transplant Cohort Study are: Patrizia Amico, John-David Aubert, Vanessa Banz, Guido Beldi, Christian Benden, Christoph Berger, Isabelle Binet, Pierre-Yves Bochud, Sanda Branca, Heiner Bucher, Thierry Carell, Emmanuelle Catana, Yves Chalandon, Sabina de Geest, Olivier de Rougemont, Michael Dickenmann, Michel Duchosal, Laure Elkrief, Thomas Fehr, Sylvie Ferrari-Lacraz, Christian Garzoni, Paola Gasche Soccal, Christophe Gaudet, Emiliano Giostra, Déla Golshayan, Karine Hadaya, Jörg Halter, Dimitri Hauri, Dominik Heim, Christoph Hess, Sven Hillinger, Hans H. Hirsch, Günther Hofbauer, Uyen Huynh-Do, Franz Immer, Richard Klaghofer, Michael Koller (Head of the data center), Bettina Laesser, Guido Laube, Roger Lehmann, Christian Lovis, Pietro Majno; Oriol Manuel, Hans-Peter Marti, Pierre Yves Martin, Michele Martinelli, Pascal Meylan, (Head, Biological samples management group), Philippe Morel, Nicolas J. Mueller (Chairman Scientific Committee), Antonia Müller, Thomas Müller, Beat Müllhaupt, Manuel Pascual (Executive office), Jakob Passweg, Klara Posfay-Barbe, Juliane Rick, Eddy Roosnek, Anne Rosselet, Silvia Rothlin, Frank Ruschitzka, Urs Schanz, Stefan Schaub, Aurelia Schnyder, Christian Seiler, Jan Sprachta; Susanne Stampf, Jürg Steiger (Head, Executive Office), Guido Stirnimann, Christian Toso, Christian Van Delden (Executive Office), Jean-Pierre Venetz, Jean Villard, Madeleine Wick (STCS coordinator), Markus Wilhelm, Patrick Yerly.
