# Erratum: Atopy as an independent predictor for long-term patient and graft survival after kidney transplantation

**DOI:** 10.3389/fimmu.2023.1175484

**Published:** 2023-03-08

**Authors:** Frontiers Production Office

**Affiliations:** Frontiers Media SA, Lausanne, Switzerland

**Keywords:** atopy, transplantation, kidney, survival, rejection, graft survival, patient survival

An omission to the funding section of the original article was made in error. The following sentence has been added: “Open access funding was provided by the University of Lausanne”.

The original version of this article has been updated.

