# Supplementary material for: Atopy as an independent predictor for long-term patient and graft survival after kidney transplantation
Source: Front Immunol. 2022 Oct 3;13:997364. doi: 10.3389/fimmu.2022.997364 (PMC9574189; doi:10.3389/fimmu.2022.997364)
Supplement: Supplementary file 1 [file DataSheet_1.docx]

**Supplementary information**

**Supplementary Table 1 | Causes of end-stage renal disease for non-atopic and atopic kidney transplant recipients**

|  | **Non-atopic** | |  | **Atopic** | |  |  |
| --- | --- | --- | --- | --- | --- | --- | --- |
|  | *N = 202* | |  | *N = 66* | |  |  |
| **Cause** | N (%) | |  | N (%) | |  | **P** |
| Congenital | 4 | (2.0%) |  | 0 | (0.0%) |  | 0.56 |
| DM nephropathy | 21 | (10.4%) |  | 5 | (7.6%) |  |  |
| GN | 55 | (27.2%) |  | 22 | (33.3%) |  |  |
| Hereditary non-PCKD | 4 | (2.0%) |  | 1 | (1.5%) |  |  |
| Interstitial nephritis | 5 | (2.5%) |  | 4 | (6.1%) |  |  |
| Hypertensive nephroangiosclerosis | 26 | (12.9%) |  | 7 | (10.6%) |  |  |
| PCKD | 41 | (20.3%) |  | 11 | (16.7%) |  |  |
| Previous GF | 6 | (3.0%) |  | 0 | (0.0%) |  |  |
| Reflux/Pyelonephritis | 14 | (6.9%) |  | 6 | (9.1%) |  |  |
| Other | 14 | (6.9%) |  | 6 | (9.1%) |  |  |
| Unknown | 12 | (5.9%) |  | 4 | (6.1%) |  |  |

DM, diabetic nephropathy; GN, glomerulonephritis; PCKD, polycystic kidney disease; GF, graft failure; P, p-value.

**Supplementary Table 2 | Transplantation surgery-related complications**

|  | **Non-atopic** | |  | **Atopic** | |  |  |
| --- | --- | --- | --- | --- | --- | --- | --- |
|  | **N** | **(%)** |  | **N** | **(%)** |  | **P** |
| **Overall complications** | *N = 202* | |  | *N= 66* | |  |  |
|  |  |  |  |  |  |  |  |
| Number of patients with complication | 87 | (43.1%) |  | 33 | (50.%) |  | 0.33 |
|  |  |  |  |  |  |  |  |
| **Causes of complications** | *N = 139* | |  | *N= 51* | |  |  |
|  |  |  |  |  |  |  |  |
| Lymphocele | 32 | (23.0%) |  | 14 | (27.5%) |  | 0.37 |
| Obstruction | 18 | (13.0%) |  | 5 | (9.8%) |  |  |
| RA stenosis | 9 | (6.5%) |  | 7 | (13.7%) |  |  |
| RAT | 1 | (0.7%) |  | 0 | (0.0%) |  |  |
| Tpx-related re-surgery | 36 | (25.9%) |  | 8 | (15.7%) |  |  |
| Urine leak | 7 | (5.0%) |  | 3 | (5.9%) |  |  |
| SSI | 1 | (0.7%) |  | 0 | (0.0%) |  |  |
| Other | 35 | (25.2%) |  | 14 | (27.5%) |  |  |

RA, renal artery; RAT, renal artery thrombosis; SSI, surgical site infection; Tpx, transplant; P, p-value.

**Supplementary Table 3 | Characteristics of non-atopic and atopic kidney transplant recipients stratified by recipient’s age**

|  | **< 50 years old** | | **≥ 50 years old** | |  |
| --- | --- | --- | --- | --- | --- |
|  | *N = 94* | | *N = 174* | |  |
| **Variable** | **N (% or IQR)** | | **N (% or IQR)** | | **P** |
| Recipient age (median; IQR) | 37.8 | (16.0) | 61.8 | (9.0) | **< 0.001** |
| Recipient gender M | 61 | (64.9%) | 141 | (81.0%) | **0.05** |
| Positive phadiatop | 33 | (35.1%) | 33 | (19.0%) | **0.005** |
| IgE total (median; IQR) | 23.0 | (89.4) | 18.2 | (64.5) | 0.12 |

N, number of patients; Ig, Immunoglobulin. P-values were computed using a Mann-Whitney test for continuous variables and a Chi-Square test for proportions.


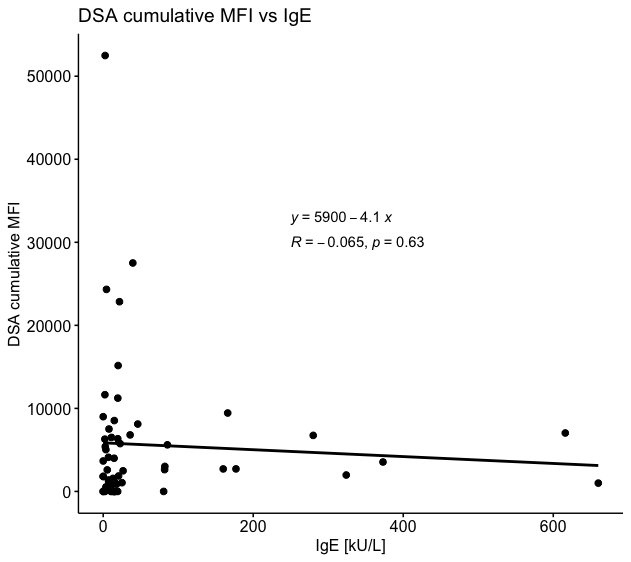


**Supplementary figure 1** | **Correlation between total IgE and preformed cumulative DSA.** The line represents the fitted linear regression model.


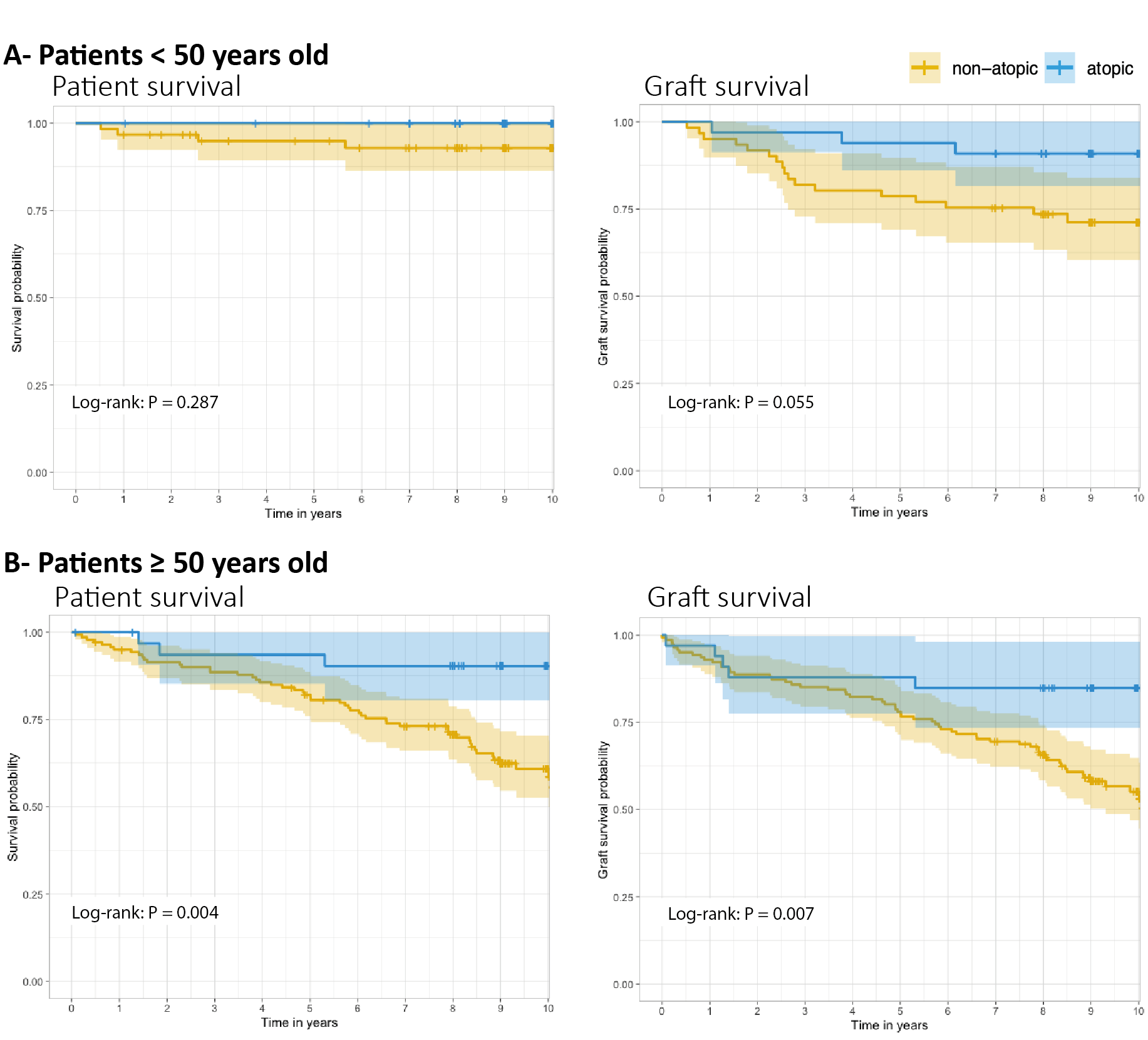


**Supplementary figure 2** | **Kaplan-Meier survival analysis stratified by recipient’s age (cut-off at 50 years-old).** Patient and graft survival for under (A) and over (B) 50-year-old patients. The calculated p-values were obtained by the log-rank test (univariate analysis). 95% confidence intervals are represented according to the group color.
