# Supplementary figures and images for: Atopy as an independent predictor for long-term patient and graft survival after kidney transplantation
Source: Front Immunol. 2022 Oct 3;13:997364. doi: 10.3389/fimmu.2022.997364 (PMC9574189; doi:10.3389/fimmu.2022.997364)

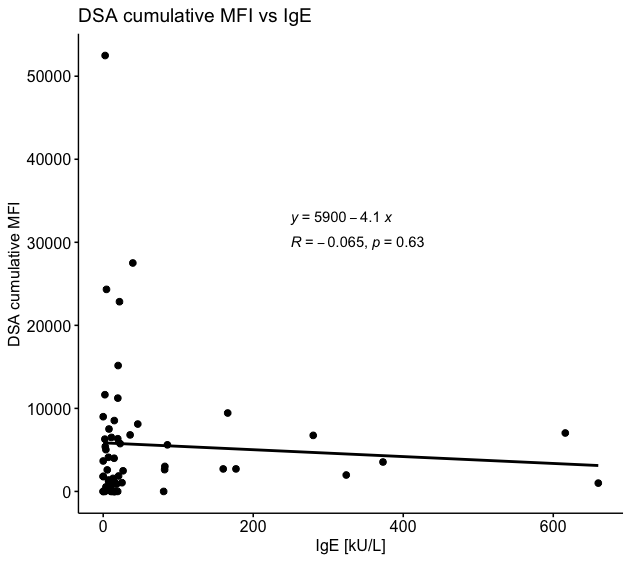

Supplement: Supplementary file 2 [file Image_1.png]

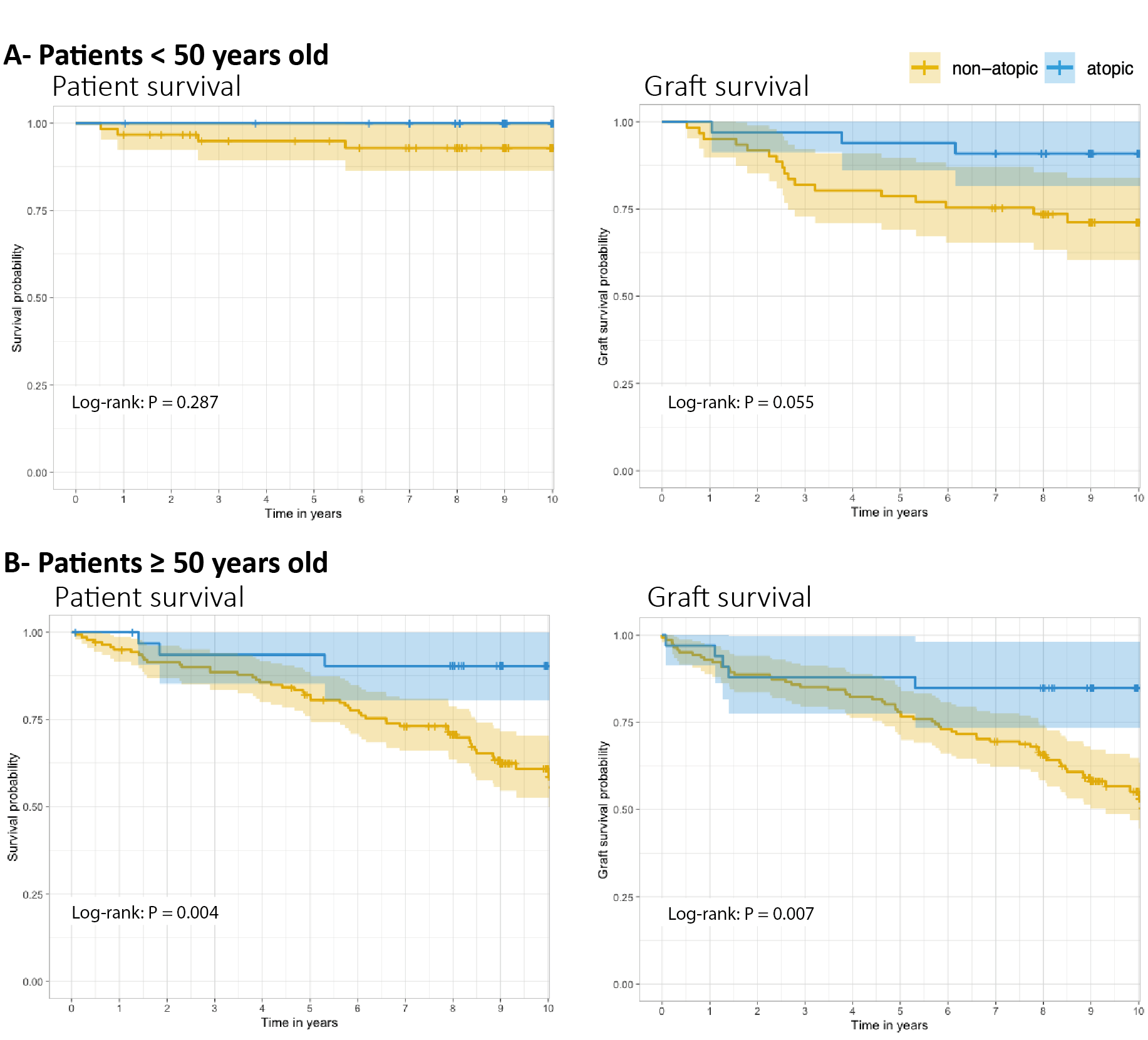

Supplement: Supplementary file 3 [file Image_2.png]
